# Glucagon-Like Peptide-1 (GLP-1) Receptor Agonists: Exploring Their Impact on Diabetes, Obesity, and Cardiovascular Health Through a Comprehensive Literature Review

**DOI:** 10.7759/cureus.68390

**Published:** 2024-09-01

**Authors:** Khalid Hamed, Mohammed N Alosaimi, Bashaer A Ali, Atheer Alghamdi, Taif Alkhashi, Salman S Alkhaldi, Nawaf A Altowarqi, Hayat Alzahrani, Abdullah M Alshehri, Rami K Alkhaldi, Khalid W Alqahtani, Nehal H Alharbi, Hanan F Alhulayfi, Shuruq Y Sharifi, Ibrahim M Dighriri

**Affiliations:** 1 Department of Clinical Toxicology, Umm Al-Qura University, Mecca, SAU; 2 Department of Pharmacy, King Abdulaziz Specialist Hospital, Ta'if, SAU; 3 Department of Pharmacy, Nahdi Medical Company, Jeddah, SAU; 4 College of Pharmacy, Taif University, Ta'if, SAU; 5 Department of Pharmacy, Rania General Hospital, Ta'if, SAU; 6 Department of Pharmacy, Dr. Sulaiman Al Habib Medical Group, Riyadh, SAU; 7 Department of Pharmacy, Tamer Group, Riyadh, SAU

**Keywords:** hba1c, exenatide, liraglutide, dulaglutide, semaglutide, glp-1 agonist

## Abstract

Glucagon-like peptide-1 receptor agonists (GLP-1-RAs) are a novel class of medications promising for treating type 2 diabetes mellitus (T2DM) and obesity-related conditions such as cardiovascular disease (CVD) and non-alcoholic fatty liver disease (NAFLD). This comprehensive literature review examines available research on these medications, focusing on their mechanisms of action, clinical effectiveness, safety profiles, and socioeconomic implications. A comprehensive search was performed using the PubMed, EMBASE, and Cochrane Library databases. Although initially developed for glucose management, these drugs have also demonstrated efficacy in promoting weight loss and reducing the risk of CVD. GLP-1-RAs function similarly to naturally occurring incretins. They stimulate insulin secretion in response to glucose levels, inhibit glucagon release, delay stomach emptying, and generate a sense of fullness via brain pathways. Head-to-head clinical studies have indicated that GLP-1-RAs outperform conventional antidiabetic medicines in terms of glycemic management and weight reduction. According to cardiovascular outcome studies, various drugs in this category have been found to reduce the frequency of severe adverse cardiovascular events. A common side effect is gastrointestinal toxicity, which can be mitigated by gradually increasing the dose. Personalized treatment is likely because the effectiveness, safety, and dose regimens of currently available GLP-1-RAs differ. GLP-1-RAs are a superior choice for patients with T2DM, especially those who already have CVD or require weight-control support. The high cost of these drugs creates hurdles to access and fair healthcare. Current research mainly focuses on increasing therapeutic uses and producing orally delivered medicines with greater potency and bioavailability. Integrating GLP-1-RAs into clinical practice can enhance patient outcomes and reduce the community burden of cardiometabolic disease.

## Introduction and background

The global prevalence of obesity has reached alarming levels, affecting more than 650 million adults as of 2016 [1]. According to the WHO, the worldwide prevalence of obesity nearly tripled between 1975 and 2016, with 39% of adults aged 18 years and over being overweight and 13% obese in 2016 [2]. This epidemic significantly increases the risk of various comorbidities, including type 2 diabetes mellitus (T2DM), cardiovascular disease (CVD), certain types of cancer, and kidney disease [3-6].

Traditional approaches to weight management, such as dietary modifications and increased physical activity, often yield limited results, particularly for individuals with severe obesity [7]. These approaches typically result in a 5-10% weight loss, which, while beneficial, may be insufficient for many patients with obesity-related comorbidities [8]. Moreover, long-term adherence to lifestyle changes remains a significant challenge, with many individuals regaining lost weight within 1-5 years [9]. This limitation underscores the need to explore alternative therapeutic options that can provide more substantial and sustainable weight loss.

In this context, glucagon-like peptide-1 receptor agonists (GLP-1-RAs) have emerged as a promising class of medications. Initially developed for the treatment of T2DM, these drugs have demonstrated significant potential in aiding weight regulation [10,11]. GLP-1-RAs are synthetic analogues of the naturally occurring incretin hormone GLP-1, which plays a crucial role in glucose homeostasis and appetite regulation [12].

The historical development of GLP-1-RAs dates back to the 1980s, when the incretin effect was first observed [13]. The first GLP-1-RA, exenatide, was approved by the FDA in 2005 for the treatment of T2DM [14]. Since then, several other GLP-1-RAs have been developed, including liraglutide, dulaglutide, and semaglutide, each with unique pharmacokinetic profiles and administration schedules [15].

GLP-1-RAs offer dual benefits: improved glycemic control and potential weight reduction [16]. This combination is particularly advantageous for individuals who are overweight or obese, especially those with significant excess weight. In a landmark development, liraglutide (Saxenda) was approved in 2020 for use in adolescents aged 12-17 years with a body weight above 60 kg and an initial BMI corresponding to ≥30 kg/m² for adults [17]. This approval was based on clinical trials demonstrating the safety and efficacy of liraglutide in this age group, offering a new treatment option for adolescents with obesity who have not responded adequately to lifestyle interventions alone [18]. It is important to note that GLP-1-RAs are not suitable for all types of obesity. They are typically indicated for individuals with a BMI ≥30 kg/m², or ≥27 kg/m² with at least one weight-related comorbidity, who have not achieved sufficient weight loss through diet and exercise alone [19]. The use of these medications in specific populations, such as adolescents, requires careful consideration of potential risks and benefits.

This comprehensive review aims to investigate the historical development of GLP-1-RA-based drugs, their mechanisms of action, therapeutic efficacy, safety profiles, tolerability, potential side effects, long-term outcomes, and future innovation trends. By synthesizing current knowledge and providing new insights into GLP-1-RA-dependent treatments for metabolic disorders and obesity, this study seeks to contribute to the evolving landscape of metabolic health management.

## Review

Methodology

This comprehensive review on the effects of GLP-1-RAs on diabetes and obesity was conducted using a structured approach to identify and synthesize relevant literature. This method ensures a thorough and up-to-date analysis of available information on GLP-1-RA-based medications.

Literature Search

The literature review involved a comprehensive search of reputable medical databases. The databases used included PubMed, EMBASE, and the Cochrane Library. The search strategy incorporated key terms such as "GLP-1," "GLP-1 receptor agonists," "weight loss," "obesity," "type 2 diabetes," "mechanism of action," "clinical efficacy," "safety," and "future trends." To ensure the inclusion of the most current information, we primarily focused on literature published within the last 20 years (2004-2024), although seminal papers from earlier dates were included when relevant to the historical context or fundamental understanding of GLP-1-RAs.

Inclusion Criteria

To obtain a comprehensive understanding of GLP-1-RAs, a range of research approaches and publishing formats were utilized. This included randomized controlled trials (RCTs), widely regarded as the gold standard for assessing therapeutic interventions, and systematic reviews and meta-analyses, which synthesized data from multiple studies. We also examined observational studies that gathered empirical data on the effects of treatment on patient outcomes. Furthermore, a thorough review of clinical practice recommendations was conducted to fully grasp the most effective procedures and expert viewpoints on the use of GLP-1-RA drugs. Studies were included if they provided detailed and relevant information on various aspects of GLP-1-RA treatment within the last 20 years. These aspects include functional methods, clinical effectiveness, safety attributes, and their significance in treating T2DM and weight management.

Exclusion Criteria

Studies that did not explicitly study GLP-1-RAs or their therapeutic uses were excluded. Publications lacking in-depth information and analysis of essential aspects of GLP-1-RAs, such as mechanisms of action, clinical effectiveness, safety profiles, relevance to T2DM treatment, and weight control, were omitted. Furthermore, the evaluation was restricted to papers published in English to ensure accessibility and uniformity throughout the review process. Using these exclusion criteria, the assessment effectively rejected irrelevant or poor sources of necessary information, allowing for a more specific and comprehensive review of the relevant literature on GLP-1-RA therapy.

Data Extraction and Synthesis

Relevant materials from various sources were carefully chosen and incorporated throughout the literature to ensure accuracy. This comprehensive approach examines the historical context, mechanisms of action, clinical efficacy in key therapeutic areas, safety profiles, and potential future breakthroughs and applications of GLP-1-RA-based drugs.

History of GLP-1-RAs

Early Identification of GLP-1-RAs

The discovery and development of GLP-1-RAs represent a significant milestone in the treatment of metabolic disorders. The journey began in the 1960s with the formulation of the incretin hypothesis, which proposed that intestinal hormones stimulate insulin secretion. This led to the identification of GLP-1 as a product of proglucagon gene expression in 1983 [20].

A crucial breakthrough came in 1987 when researchers observed the insulinotropic effects of GLP-1 in humans, confirming its potential for treating T2DM [21]. Initially, native GLP-1 was administered intravenously in clinical trials due to its peptide nature. However, this approach faced significant challenges that initially hindered the development of GLP-1-based therapies: Endogenous GLP-1 is quickly broken down by the enzyme dipeptidyl peptidase-4 (DPP-4), resulting in a very short half-life of approximately 2 minutes [22]. This rapid degradation meant that continuous infusion would be required for therapeutic effect, which was impractical for long-term treatment. Attempts to administer native GLP-1 subcutaneously or orally were unsuccessful due to poor absorption and rapid degradation before reaching the circulation in sufficient quantities [23]. These challenges led researchers to focus on developing GLP-1 analogs that were resistant to DPP-4 degradation, had improved bioavailability, and could be administered less frequently. This shift in focus marked a significant turning point in GLP-1-RA development [11].

The first successful GLP-1-RA, exenatide, was derived from the saliva of the Gila monster and was found to be resistant to DPP-4 degradation. It was approved by the FDA in 2005 for subcutaneous administration, initially twice daily [24]. This was followed by the development of human GLP-1 analogs like liraglutide, which incorporated fatty acid molecules to extend their half-life and allow for once-daily dosing [25]. Subsequent research has led to the development of long-acting GLP-1-RAs that can be administered weekly, further improving patient convenience and potentially enhancing treatment adherence [15]. Understanding these early challenges and the subsequent innovations has been crucial in shaping the current landscape of GLP-1-RA therapeutics, leading to more effective and patient-friendly treatment options for T2DM and obesity.

Key Developments of GLP-1-RAs

The development of GLP-1-RAs has seen significant advancements since the early 2000s, marking a new era in the treatment of T2DM and obesity [11]. Clinical research on GLP-1-RAs began in earnest in the early 1990s, with researchers exploring their potential for glucose regulation and weight management [26]. The first major breakthrough came in 2005 when the U.S. FDA approved exenatide (Byetta) as the first GLP-1-RA for the treatment of T2DM [27]. This twice-daily injectable medication represented a significant step forward in diabetes management.

In 2009, liraglutide (Victoza) received FDA approval for once-daily injection in T2DM treatment, offering improved convenience for patients [28]. The following year, in 2010, a long-acting formulation of exenatide (Bydureon) was developed, allowing for once-weekly dosing and further enhancing patient adherence [29]. The year 2014 saw the approval of two new GLP-1-RAs: dulaglutide (Trulicity) and albiglutide (Tanzeum), both offering once-weekly dosing options [15]. These additions expanded the range of treatment options available to patients and healthcare providers.

A significant milestone was reached in 2014 when liraglutide, under the brand name Saxenda, was approved by the FDA for chronic weight management in adults with obesity (BMI ≥30 kg/m²) or overweight (BMI ≥27 kg/m²) with at least one weight-related comorbidity [30]. It is important to note that Saxenda is a prescription medication and not available over-the-counter. The approval of Saxenda marked the first time a GLP-1-RA was officially indicated for weight management, broadening the therapeutic applications of this drug class. In 2017, semaglutide (Ozempic) was approved for T2DM treatment, demonstrating enhanced efficacy in both glycemic control and weight loss compared to earlier GLP-1-RAs [31]. This was followed by the groundbreaking approval of oral semaglutide (Rybelsus) in 2019, marking the first oral GLP-1-RA to be made available (Table 1) [32].

**Table 1:** Key developments of GLP-1-RAs. GLP-1-RAs: Glucagon-like peptide-1 receptor agonists.

Year	GLP-1-RAs Development
1993	Initiation of clinical studies with GLP-1-RAs analogs [26]
2005	Exenatide (Byetta) becomes the first GLP-1-RAs approved [11]
2009	Liraglutide (Victoza) is approved for once-daily injection [25]
2012	Extended-release Exenatide (Bydureon) is approved for weekly dosing [33]
2014	Approvals of Dulaglutide (Trulicity) and Albiglutide (Tanzeum) [15]
2016	Liraglutide (Saxenda) is approved for weight management [30]
2017	Semaglutide (Ozempic) was noted for enhanced efficacy in control and weight loss [31]
2019	Oral Semaglutide (Rybelsus) was approved, a first for oral dosing [32]

Evolution and Refinement

GLP-1-RAs have been extensively researched and developed over the last two decades and are increasingly included in the treatment guidelines for metabolic illnesses. The growing accessibility and diversity of these treatments offer several benefits, including convenient administration, the ability to adjust dosages for personalized medicine, the promotion of weight loss, and a decrease in the burden of therapy [34]. Additionally, they aim to achieve optimal control of glucose levels. Owing to their inadequate performance, several agonists, such as albiglutide, have been withdrawn from the market. However, each failure served as a catalyst for further research into the creation of more potent and effective GLP-1-RAs [34,35].

Current Landscape

As of 2024, exenatide, liraglutide, dulaglutide, semaglutide, and lixisenatide are the globally available GLP-1-RAs. Notably, seven GLP-1-RAs have been approved in Europe, and almost all have received approval from the United States of America. They are prescription medications and are not available over the counter (OTC) [36,37].

Ongoing Research

Ongoing research endeavors to create novel GLP-1-RAs exemplify the scientific community's dedication to meeting unmet needs and enhancing the well-being of people with T2DM and obesity worldwide. This development is evident, even though there are challenges in maintaining stability and continuous quality control throughout the manufacturing process [34,38].

Mechanism of action

Due to their independent activity, GLP-1-RAs can modulate glucose levels, hunger, and energy balance via various channel mechanisms. Abundant in the pancreas, stomach, brain, and cardiovascular systems, GLP-1-RAs are the primary mechanisms responsible for controlling the activity of these organs [39]. The therapeutic success of GLP-1-RAs in managing T2DM and obesity has been attributed to their diverse modes of action across various physiological systems. When administered to the pancreas, GLP-1-RAs can boost glucose-dependent insulin release from β-cells, lowering the likelihood of hypoglycemia. In hyperglycemic situations, they also limit the release of glucagon from α-cells, which improves glucose homeostasis by reducing glucose synthesis in the liver [40,41].

Furthermore, it is worth noting that these agonists actively encourage the proliferation of β-cells and inhibit apoptosis, which has the potential to slow the onset of T2DM [42]. Delayed stomach emptying is a known gastrointestinal effect. This delay helps reduce postprandial glucose spikes and increases satiety, thereby reducing food consumption [43,44]. Additionally, a few studies suggest a decrease in the intestinal absorption of glucose, which in turn aids glycemic management. GLP-1-RAs in the hypothalamus and brainstem are responsible for regulating food intake and energy expenditure in the central nervous system. When activated, these receptors increase satiety and decrease feelings of hunger [45,46].

**Figure 1:**
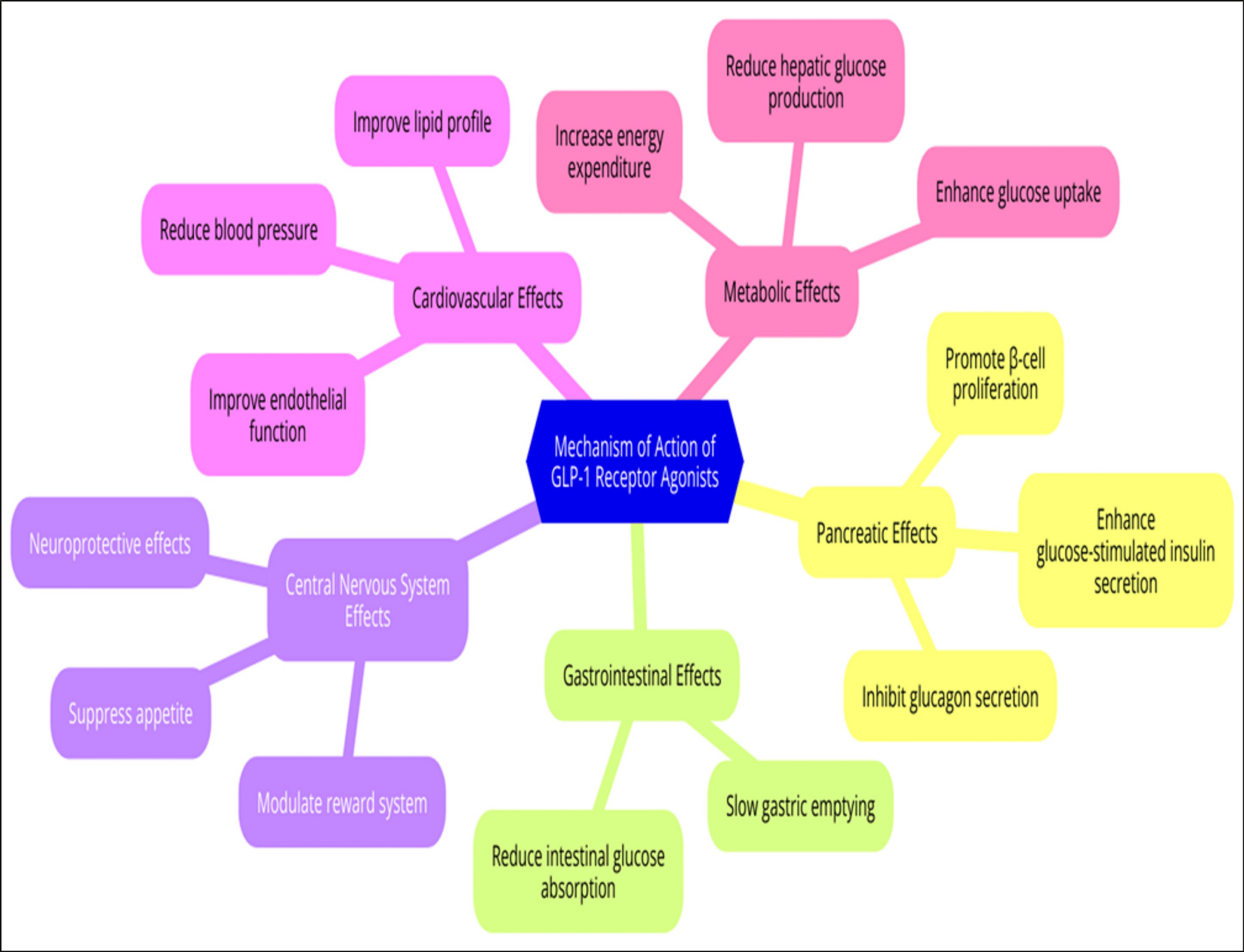
Mechanism of action of GLP-1-RAs. GLP-1-RAs: Glucagon-like peptide-1 receptor agonists.

Moreover, these receptors can influence the mesolimbic dopamine pathway, which might address reward-driven overeating in obese individuals [47]. Furthermore, data suggest neuroprotective benefits that could improve cognitive function and prevent neurodegenerative illnesses [48]. Some cardiovascular advantages of GLP-1-RAs include a slight decrease in systolic blood pressure, improvement in endothelial function, and beneficial effects on lipid profiles, such as a reduction in triglycerides and low-density lipoprotein (LDL) cholesterol [49-51]. Regarding metabolism, these medications improve insulin sensitivity and glucose absorption in peripheral tissues, reduce glucose synthesis in the liver via a variety of mechanisms, and have the potential to increase energy expenditure, which may lead to weight reduction (Figure 1) [52,53].

GLP-1-RAs are attractive treatments for metabolic disorders, including T2DM and obesity, owing to the intricate interplay between various pathways contributing to the diverse effects of these agonists. Examples of such ailments include obesity and T2DM. However, further investigation is required into the complicated relationship between homeostatic and non-homeostatic eating control in response to GLP-1-RAs [12,53].

Efficacy in clinical use

GLP-1-RAs have shown substantial efficacy in clinical trials and real-world studies, particularly in terms of glycemic control, weight management, and cardiovascular risk [35,54].

Glycemic Control

Every clinical study and meta-analysis of GLP-1-RAs has consistently shown high efficacy in regulating glucose levels. These medications have resulted in HbA1c reductions ranging from 0.8% to 1.8% in clinical trials [55]. Additionally, a comprehensive review showed that the average decrease in HbA1c level was 1.01% compared with placebo [56]. GLP-1-RAs significantly affect postprandial glucose excursions by affecting gastric emptying and decreasing glucagon levels, effectively reducing glucose levels during fasting and after food consumption. Comparative tests have demonstrated that newer GLP-1-RAs, including semaglutide, offer superior glycemic control compared with previous medications. An illustrative instance is the SUSTAIN-7 study, which revealed that semaglutide yielded significantly more significant reductions in HbA1c levels than dulaglutide (-1.8% vs. -1.4% for the highest doses) [57]. This highlights ongoing progress in these medications [30,58].

Weight Management

GLP-1-RAs have become a feasible treatment option for individuals who are overweight or obese and have T2DM. These drugs have significant and enduring benefits in terms of weight loss. STEP-1 research showed that semaglutide 2.4 mg led to an average weight reduction of 14.9% over 68 weeks [59]. A previous study has shown significant decreases in body weight, ranging from 5% to 15% of the initial body weight [30]. Additionally, a 14.9% weight reduction was observed over 68 weeks. A meta-analysis of 12 RCTs found that the experimental groups achieved a significantly greater mean weight reduction of -7.1 kg than the control groups [60]. Semaglutide showed the greatest efficacy in terms of weight reduction among the GLP-1-RAs. Research has shown that the decrease in weight achieved using GLP-1-RAs may be maintained for extended durations, with some individuals continuing to lose weight even after the first year of medical use [61]. The efficacy of GLP-1-RAs in managing obesity-related complications in individuals with T2DM is underscored by their long-term effectiveness in weight management [62].

Cardiovascular Benefits

Multiple GLP-1-RAs agonists have shown significant cardiovascular benefits in extensive outcome trials, marking an essential advancement in T2DM management. The LEADER study showed that liraglutide reduced the risk of Major Adverse Cardiovascular Events (MACE) by 13% in individuals with T2DM and high cardiovascular risk compared to placebo [63]. However, the SUSTAIN-6 trial showed a more remarkable decrease in the risk of MACE with semaglutide [64]. These medications have shown potential in reducing the occurrence of nonfatal myocardial infarctions, nonfatal strokes, and cardiovascular mortality. An extensive meta-analysis of trials on cardiovascular outcomes showed that GLP-1-RAs reduced overall mortality and cardiovascular mortality by 12% and 14%, respectively [65]. This finding emphasizes the capacity of these drugs to improve the total lifespan of high-risk individuals. Additionally, some GLP-1-RAs have been shown to improve renal protection, as indicated by a reduction in the progression of albuminuria and a decrease in the occurrence of renal events. The cardiovascular and renal benefits, combined with their impact on glucose levels and weight loss, make GLP-1-RAs a versatile therapeutic option for patients with T2DM, particularly for those at an increased risk of cardiovascular problems [16,66].

Additional Metabolic Benefits

GLP-1-RAs have been shown to benefit cardiovascular risk factors in addition to their primary functions of reducing glucose levels and facilitating weight loss. These medications are associated with significant reductions in systolic blood pressure, often ranging from 2-5 mmHg [67,68]. These decreases are not just small in magnitude but also have substantial therapeutic relevance. Reducing blood pressure may decrease the overall risk of CVD, particularly in those with hypertension [51,68]. Furthermore, it is worth noting that many GLP-1-RAs have a positive effect on lipid profiles, including reductions in triglycerides and modest increases in HDL cholesterol [51,69]. Although the changes in lipid indicators may be small, they can provide further cardiovascular benefits when combined with other positive effects of GLP-1-RAs. The enhancements in blood pressure and lipid profiles, in addition to their primary effects on glycemic control and body weight, augment the potential of GLP-1-RAs as comprehensive therapeutic choices for patients with T2DM, especially for those with multiple cardiovascular risk factors in their medical history [51,69].

Comparative Efficacy

GLP-1-RAs have shown remarkable performance compared to other antidiabetic medications, making them potentially valuable therapeutic options for managing T2DM. These medicines have demonstrated efficacy in glycemic control and weight loss, which is superior to or equal to that of many other diabetic therapies. GLP-1-RAs differ from sulfonylureas and insulin because they may aid in weight loss and have a low risk of hypoglycemia [57]. Semaglutide, in particular, is a very successful therapy within the GLP-1-RAs category. It has shown superior glycemic control and weight reduction efficacy compared with other GLP-1-RAs. This benefit surpasses its category, as seen in the SUSTAIN-8 research, where semaglutide significantly reduced HbA1c levels and weight loss more than canagliflozin, an SGLT2 inhibitor [70]. In the individualized treatment of T2DM, these studies on the effectiveness of GLP-1-RAs, especially newer ones such as semaglutide, emphasize their potential as effective strategies, particularly for patients who prioritize weight loss and cardiovascular risk reduction in addition to glycemic management [70,71].

GLP-1-RAs are versatile therapeutic agents that can be used to treat the intricate interplay of metabolic disorders. These medications are not only beneficial for controlling blood sugar levels and reducing weight but are also essential for treating metabolic diseases. As they target several aspects of metabolic health, they are valuable tools for managing obesity and T2DM [6,71].

Safety and tolerability

Although GLP-1-RAs have shown considerable therapeutic advantages, it is critical to assess their safety and tolerability before use in clinical settings.

Most Common Side Effects

The most frequently reported side effect of GLP-1-RAs is gastrointestinal problems. Symptoms include nausea, vomiting, diarrhea, and stomach discomfort [72]. The occurrence of these unfavorable outcomes can vary from 12% to 66%, depending on the substance and dosage used [73]. Patients on tirzepatide experienced nausea in 12-18% of cases, but those taking semaglutide reported nausea in 20-24% of cases. Injection site reactions, such as redness, itching, or swelling, may occur in 1-5% of patients. These reactions are generally mild and transient [15]. Headache is a common adverse effect, usually mild in intensity and short-lived. According to estimates, this condition affects 5-10% of the population [74]. Although these side effects are common, they can often be managed without discontinuation of the medication [15,74].

Mitigation Strategies

GLP-1-RAs are often associated with undesirable gastrointestinal side effects. Nonetheless, these effects frequently diminish over time as patients become accustomed to the medication. To mitigate the severity of these negative outcomes and enhance medication acceptability, it is advisable to start with a low dose and gradually increase it over several weeks [75,76]. Furthermore, patients may find relief by modifying their diet, such as by eating smaller, more frequent meals throughout the day. Using these strategies, patients can successfully manage the gastrointestinal symptoms associated with GLP-1-RAs while also improving their treatment experience [72,75].

Hypoglycemia Risk

The risk of hypoglycemia associated with GLP-1-RAs monotherapy is modest [31,77]. A meta-analysis of clinical studies using exenatide, liraglutide, and albiglutide found that the incidence of hypoglycemia varied from 0 to 1.5% when these medications were administered alone [78]. However, when combined with insulin or sulfonylureas, GLP-1-RAs are associated with increased vulnerability to hypoglycemia [36,77]. In the DURATION-6 study, 6% of patients who received exenatide and 5% of those who received liraglutide experienced mild hypoglycemia when combined with sulfonylureas [74]. The SUSTAIN-6 study, which examined the cardiovascular safety of semaglutide, found that 1.4% of patients receiving semaglutide experienced severe hypoglycemia compared with 1.5% of patients in the placebo group [31,77]. The LEADER study yielded comparable results as it evaluated the cardiovascular safety of liraglutide. The incidence of severe hypoglycemia was 1.3% in the liraglutide group and 1.5% in the placebo group, revealing that although the risk of low blood sugar levels is normally modest with GLP-1-RAs, care should be taken when taking these drugs in conjunction with other glucose-lowering therapies that are known to induce hypoglycemia [79].

Rare But Serious Adverse Events

Although GLP-1-RAs are typically well-tolerated, there have been concerns about the occurrence of rare but serious side effects. Nonetheless, several studies and meta-analyses have failed to demonstrate a substantially high risk of pancreatitis. Early investigations suggested an increased vulnerability to pancreatitis [80]. In the LEADER study, the incidence of pancreatitis was 0.4% among those treated with liraglutide, compared to 0.5% among those administered a placebo [79]. Research on rats has indicated an increased risk of medullary thyroid cancer, which is another reason for caution [25]. However, clinical research in humans has not shown any evidence of a higher risk [81]. Several studies have shown increased vulnerability to gallbladder-related disorders, such as cholelithiasis and cholecystitis [82]. A meta-analysis found a link between the use of GLP-1-RAs and an increased risk of gallbladder or biliary problems [83]. When administering GLP-1-RAs, healthcare practitioners should be aware of these important, albeit uncommon, side effects and carefully monitor patients in accordance with medical standards.

Special Populations

Assessing patients’ kidney and liver function, as well as their age, is critical in determining whether to employ GLP-1-RAs in certain populations. Most GLP-1-RAs can be administered to patients with moderate-to-severe renal impairment without adjusting their dosage [57]. It is recommended that patients with considerable renal impairment exercise caution, and some medications require a dosage adjustment or may not be appropriate for use. Similarly, for individuals with hepatic impairment, dosage modification is often unnecessary for mild to moderate impairment, although studies on severe hepatic impairment are limited. There is no need to change dosages in older people based solely on age. However, owing to the possibility of impaired renal function and increased susceptibility to gastrointestinal side effects, caution is advised [84]. When prescribing GLP-1-RAs, medical practitioners should carefully consider the parameters listed above to ensure that these medications are used safely and effectively in the described categories.

Considering the increased use of GLP-1-RAs in a broader spectrum of patients and for longer periods, it is critical to fully understand their long-term safety repercussions [85]. Pancreatitis, medullary thyroid cancer, and gallbladder-related disorders are serious side effects that may appear only after chronic use in larger groups of individuals [80,85]. This is particularly important, given the potential for these adverse events to occur. To enhance the safety of GLP-1-RAs in clinical practice, it is critical to carefully select patients and adequately manage gastrointestinal side effects, despite the medications' typically excellent tolerance [80]. Healthcare providers should be aware of the possible dangers associated with these drugs, and careful monitoring is recommended for patients with renal or hepatic impairment, as well as for the elderly. To evaluate the safety of GLP-1-RAs in various patient groups and to determine the best way to use them to treat T2DM, it is critical to continue monitoring their effects and conducting long-term studies.

Differences between GLP-1-RAs

There are significant disparities among medications in terms of effectiveness, safety profiles, dosage regimens, and pharmacokinetic features. Although GLP-1-RAs have a similar mechanism of action, there are substantial differences between the various medications in this family. Understanding these distinctions is critical for providing optimal patient care and for developing tailored treatment options (Table 2).

**Table 2:** Summarizing the distinctions between GLP-1-RAs in different categories. GLP-1-RAs: Glucagon-like peptide-1 receptor agonists; GI: Gastrointestinal.

Category	Aspect	Details
Efficacy Differences	Glycemic Control	Research has demonstrated that semaglutide is more effective than other GLP-1-RAs in decreasing HbA1c levels [58]. The SUSTAIN-7 study found that semaglutide 1.0 mg reduced HbA1c by 1.8% more than dulaglutide 1.5 mg, which reduced it by just 1.4% [58].
	Weight Loss	In clinical trials, semaglutide had the most dramatic weight loss results, with reductions of up to 15% of the individual's baseline body weight [59]. Both liraglutide and dulaglutide have been proven to cause weight reduction, generally between 3 and 9 percent of the person's starting weight [30].
	Cardiovascular Outcomes	Liraglutide, semaglutide, and dulaglutide improve cardiovascular health [79]. Semaglutide reduced MACE by 26%, while liraglutide decreased it by 13%. The degree of the influence may vary [79,86].
Safety and Tolerability	Gastrointestinal Side Effects	The prevalence and severity of gastrointestinal (GI) side effects caused by GLP-1-RAs may vary [72]. In compared to dulaglutide, they had a somewhat greater incidence of nausea and vomiting [73]. Semaglutide and liraglutide are also linked to this condition.
	Injection Site Reactions	All agents are normally moderate; however, the frequency might vary substantially amongst agents [15].
	Pancreatitis and Thyroid Safety	It has been shown that there are no major differences between agents [87]
Pharmacokinetics and Dosing	Half-life and Dosing Frequency	Exenatide is taken twice a day and has a short duration of effect (4-6 hours) [88]. Liraglutide is suggested to be taken one daily [89]. Semaglutide, dulaglutide, and exenatide are long-acting medications that are administered once weekly [15].
	Route of Administration	Most GLP-1-RAs are given subcutaneously [15]. Semaglutide is now the sole oral GLP-1-RAs available on the market [32].
	Size and Structure	Exenatide and liraglutide are medicines created from modified GLP-1-RAs or exendin-4, providing more examples of such medications [90]. Both dulaglutide and semaglutide have bigger molecules, which results in a longer half-life [91].
Cost Differences		Newer medications, such as semaglutide, are frequently more expensive than older choices, such as liraglutide and exenatide [92]. Cost concerns are likely to have an influence on the process of making treatment choices and access to therapy [73,93].
Special Population	Renal Impairment	Exenatide doses must be adjusted for patients with mild renal impairment. However, it is not suggested that those with severe renal impairment use this medication [57]. Individuals with kidney issues, particularly those with end-stage renal illness, may be administered liraglutide, dulaglutide, and semaglutide with no further dosage adjustments [57].
Considerations	Cardiovascular Risk	Liraglutide, semaglutide, and dulaglutide have beneficial effects on the cardiovascular system and may be favored for patients at high risk of cardiovascular problems [94].

Place in treatment guidelines

T2DM Management

The ADA recommends that patients with T2DM, particularly those with established atherosclerotic cardiovascular disease (ASCVD) or a high cardiovascular risk, should utilize GLP-1-RAs as the first-line therapy in conjunction with metformin. Furthermore, GLP-1-RAs are indicated as a first-line treatment for patients seeking to reduce weight gain or encourage weight reduction. The European Association for the Study of Diabetes (EASD) and the American Diabetes Association Consensus Report recommend GLP-1-RAs as second-line therapy for most patients with T2DM after metformin. Additionally, GLP-1-RAs should be administered to individuals with proven adverse ASCVD because these drugs have been found to have cardiovascular benefits [95,96]. The American Association of Clinical Endocrinologists (AACE) and the American College of Endocrinology (ACE) have developed therapeutic strategies that prioritize GLP-1-RAs. They are not only recommended as the primary therapy in certain situations but also as an early supplement to metformin [57,95].

Cardiovascular Risk Reduction

The ADA recommends that patients with T2DM, particularly those with established ASCVD or a high cardiovascular risk, utilize GLP-1-RAs as the first-line therapy in conjunction with metformin. Furthermore, GLP-1-RAs are indicated as a first-line treatment for reducing weight gain or encouraging weight reduction [97]. The EASD and the ADA Consensus Report recommend that GLP-1-RAs be used as second-line therapy for most people with T2DM after metformin [57,96]. Furthermore, GLP-1-RAs should be administered to patients with established adult-onset chronic CVD, because these drugs have been found to have cardiovascular benefits. The AACE and ACE Guidelines recommend GLP-1-RAs as the first therapeutic algorithm. According to these guidelines, GLP-1-RAs should be considered the primary treatment choice in certain instances as well as an early addition to metformin [98,99].

Obesity Management

The Obesity Society Guidelines recommend the use of GLP-1-RAs for weight loss, particularly in patients with obesity-related comorbidities. GLP-1-RAs should be considered as a second-line therapy option following lifestyle improvements or as a first-line medicine for very obese individuals. The American Heart Association's Scientific Statement acknowledges that GLP-1-RAs have the potential to be effective in treating obesity, especially in patients with cardiovascular risk factors [97].

Combination Therapy

When single-drug treatment fails to reach glycemic targets, the ADA/EASD Consensus Report recommends adding GLP-1-RAs to current diabetes drugs, including insulin [97]. Similarly, the AACE/ACE Guidelines propose GLP-1-RAs as the optimum option for combination therapy because their mechanisms of action complement those of other antidiabetic medications [98,100].

Special Populations

The Kidney Disease Improving Global Outcomes (KDIGO) recommendations suggest GLP-1-RAs as the preferred medicine for patients with T2DM and chronic kidney disease. This is because these medications can protect the kidneys. Furthermore, the European Association for the Study of the Liver (EASL) recommends emphasizing the potential advantages of GLP-1-RAs in treating non-alcoholic fatty liver disease (NAFLD), particularly in patients with T2DM [101].

Individualized Approach

All criteria that must be followed emphasize the need to design individualized treatment plans that consider patient preferences, concurrent medical conditions, and treatment objectives [102]. Several considerations have been evaluated when determining the optimal placement of GLP-1-RAs in a patient's treatment regimen. These considerations include the desire to lose weight, the existence of heart disease, the danger of low blood sugar, and financial burden. GLP-1-RAs are becoming increasingly important in the treatment of cardiometabolic disorders, as shown by their inclusion in key recommendations for diabetes, CVD, and obesity management. The function of GLP-1-RAs in treatment algorithms is expected to change and expand as more long-term evidence is collected and new medications are launched [96,97].

Socioeconomic and healthcare implications

The use of GLP-1-RAs has significant socioeconomic and healthcare implications, influencing both individual patient treatment and the general operation of health systems.

Cost Considerations

When choosing GLP-1-RAs for diabetic therapy, cost is an important factor. These drugs are more expensive than traditional diabetic treatments, which may be a barrier for patients and the healthcare system. In a German sickness fund study, the average annual treatment costs were €6,851 for GLP-1 receptor agonists, compared to €4,895 for empagliflozin (an SGLT2 inhibitor). However, when considering these medications, it's crucial to assess their long-term effects on health outcomes and overall healthcare costs [103]. Increased glycemic and weight control reductions may offset the greater initial expenditures [104]. In the United Kingdom, liraglutide, a GLP-1-RAs, is more cost-effective than insulin glargine, with an incremental cost-effectiveness ratio of £13,228 per quality-adjusted life years (QALYs) [57]. Despite their higher cost, GLP-1-RAs may enhance patient outcomes and reduce long-term healthcare expenses; therefore, they should be considered when selecting treatment [104].

Cost-Effectiveness

Cost-effectiveness is critical when deciding whether to use GLP-1-RAs to treat diabetes. Despite the increased initial prescription costs, several studies have indicated that these drugs may be cost-effective over time. The QALYs that assess the length and quality of life improved by an intervention are popular cost-effectiveness metrics. A Norwegian cost-effectiveness analysis discovered that lixisenatide, a GLP-1-RAs, enhances QALYs while lowering lifetime healthcare expenditure compared to bolus insulin. This shows that, despite their higher initial cost, GLP-1-RAs may provide long-term financial advantages [105]. Some GLP-1-RAs have cardiovascular benefits that potentially increase their cost-effectiveness, particularly in high-risk patients. These drugs may prevent cardiovascular events and save money while improving patient outcomes. The cost-effectiveness of GLP-1-RAs varies by agent and patient group, but their long-term benefits and potential to minimize diabetes-related healthcare expenditures make them an affordable diabetes treatment option [105].

Healthcare Resource Utilization

When evaluating the effectiveness of GLP-1-RAs in diabetes therapy, it is important to assess their influence on healthcare resource utilization. These drugs may minimize hospitalization and emergency room visits, resulting in cost savings for the healthcare system. Improved glucose control and fewer diabetes-related complications make this possible [106,107]. GLP-1-RAs, which efficiently regulate blood glucose levels and weight loss, may prevent or postpone diabetes-related complications such as CVD, renal disease, and neuropathy. However, GLP-1-RAs may temporarily boost the demand for outpatient medical services [106,107]. Monitoring and managing side effects such as gastrointestinal difficulties and injection site responses may require additional outpatient visits and tests. Outpatient treatment may partly offset cost savings from fewer hospitalizations and ER visits. Improved blood sugar management and fewer complications may reduce healthcare resource consumption over time. When selecting diabetes therapies and allocating expenditures, healthcare practitioners and payers should consider GLP-1-RAs [107].

Access and Equity

Patients in resource-constrained or low-income settings may struggle to obtain GLP-1-RAs due to their high costs. The average annual cost of GLP-1-RAs can range from approximately $5,000 to $10,000 per patient in the United States [108]. In 2023, the list price for Ozempic® (semaglutide) is about $936 for a 4-week supply, which translates to roughly $12,168 per year [109]. These drugs may improve glycemic control, weight management, and cardiovascular risk, but their high cost may prevent their use [110]. This pricing barrier may raise disparities in GLP-1-RA availability, exacerbating T2DM and obesity treatment disparities, particularly in low- and middle-income countries where such medications may be entirely out of reach for most patients [111-113]. To fully achieve the promise of GLP-1-RAs in improving diabetes medication and minimizing complications, access hurdles must be addressed, and a uniform distribution across populations is ensured. Value-based pricing, patient support programs, and cooperation among healthcare providers, payers, and legislators may help overcome these barriers and increase access to promising drugs [111,114].

Public Health Impact

The widespread use of GLP-1-RAs may benefit public health by lowering the burden of metabolic diseases in healthcare systems and improving health outcomes. If GLP-1-RAs delay T2DM and obesity, their use may decrease the demand for healthcare resources [77,115]. GLP-1-RAs may enhance glycemic control, weight reduction, and cardiovascular risk, thereby preventing or delaying expensive and severe illnesses, such as CVD, renal disease, and neuropathy. This might reduce healthcare costs, enhance patient well-being, and increase productivity [115]. GLP-1-RAs may also help to prevent CVD and death by managing T2DM and obesity [31,94]. Because CVD is the leading cause of mortality globally, GLP-1-RAs may benefit public health by lowering the risk [116]. These public health benefits include removing obstacles to accessing these medicines, ensuring fair distribution, and promoting optimum usage as part of a comprehensive T2DM and obesity strategy [31,36].

Payer and Policy Implications

Increasing GLP-1-RAs use has major implications for both payers and policymakers. They must carefully balance access to potential treatments with health care expense management. Owing to the increased demand, health insurance and government payers must evaluate their reimbursement policies and formulary options for GLP-1-RAs. This may include assessing GLP-1-RAs long-term cost-effectiveness, their ability to minimize complications and healthcare usage, and reimbursement policy changes. GLP-1-RAs are costly; thus, creative cost sharing and value-based pricing strategies may be required. This is critical for the long-term viability of these drugs in healthcare [108,117]. These options might include linking payments to patient outcomes, risk-sharing with manufacturers, or tiered formularies that promote cost-effective medications. Policymakers should also consider the social benefits of GLP-1-RAs, such as reducing health inequities and enhancing the overall population health. Patient support efforts or provider education and awareness campaigns may help to overcome access challenges. Payers, lawmakers, healthcare experts, and patient advocates must collaborate in employing GLP-1-RAs to treat T2DM and obesity. This collaboration is required to develop long-term, equitable policies that maximize GLP-1-RAs benefits while limiting healthcare costs [118,119].

Workforce and Infrastructure Requirements

GLP-1-RAs are increasingly utilized to treat T2DM and obesity, which may require adjustments to healthcare staff and infrastructure to ensure patient safety. As GLP-1-RAs use increases, clinicians may need extra training to initiate and maintain treatment. This might include learning about the precise indications, doses, and side effects of various medications, as well as patient selection, monitoring, and evaluation techniques. Healthcare organizations may have to modify their infrastructure to accommodate GLP-1-RAs users [61,120]. Establishing specialist clinics or programs to manage GLP-1-RAs, implementing standardized patient education and monitoring protocols, and committing funding to electronic health record systems that track drug adherence and detect adverse events. The increasing use of GLP-1-RAs may require additional services, such as T2DM education, nutritional counseling, and behavioral support, to assist patients in optimizing results and managing adverse effects [60,121]. Competent personnel and infrastructure are required for the safe and successful use of GLP-1-RAs. This is crucial to fully realize the promise of these drugs in enhancing patient outcomes, while also lowering the cost of T2DM and obesity in healthcare systems [103,121].

Long-Term Economic Impact

GLP-1-RAs have long-term economic implications, beyond drug expenses and healthcare savings. These medications have the potential to increase productivity and reduce expenses by lowering the risk of T2DM and obesity. According to previous studies, GLP-1-RAs help to manage blood sugar, help people lose weight, and prevent cardiovascular events. These effects mitigate the long-term consequences of chronic disorders [122,123]. GLP-1-RAs may reduce impairments, workplace absenteeism, and overall efficiency. Consequently, this may benefit both individuals and society. Improved GLP-1-RAs administration may help control obesity and T2DM, thereby lowering societal expenses [37]. Productivity is reduced by high caregiver load, low quality of life, and early death due to obesity and T2DM. GLP-1-RAs may help lower societal expenses by avoiding illnesses and their impacts. To optimize the long-term economic benefits of GLP-1-RAs, a complete strategy must address access, adherence, and integration into holistic disease management programs. Policymakers, payers, and healthcare providers must collaborate to optimize GLP-1-RAs long-term economic benefits. This collaboration can improve patient outcomes, while reducing short-term expenses [124,125].

Despite their therapeutic advantages, GLP-1-RAs have complicated economic and healthcare consequences. Healthcare companies and governments must balance long-term savings and health benefits, with higher upfront expenses and access challenges. Making informed decisions and ensuring equal access to these promising treatments requires further study of their cost-effectiveness and long-term impact [125,126].

Future trends and developments

GLP-1 receptor agonists, often known as GLP-1RAs, are rapidly evolving with many interesting trends and discoveries. The primary goal of this study was to improve medication potency, duration of action, and pharmacokinetics (PK). This allows for less frequent dosing, which may result in higher efficacy [38]. Because of the positive results of oral semaglutide, there are ongoing efforts to manufacture alternative oral GLP-1RAs to boost patient compliance and convenience [15,36]. Combination medications are currently being investigated to improve glycemic control and aid in weight loss. These drugs include dual and triple agonists that specifically target several receptors, including GLP-1-RAs and glucagon [127]. Fixed-ratio combinations of GLP-1RAs with basal insulin or other antidiabetic medications are being investigated to enhance treatment options. These combinations are administered via a single injection [128]. In addition to treating T2DM and obesity, GLP-1RAs are increasingly being utilized therapeutically for NAFLD, non-alcoholic steatohepatitis (NASH) [89], neurodegenerative illnesses, and preservation of the cardiovascular and renal systems [94,129].

Personalized medical approaches, such as pharmacogenomics and biomarker-guided therapy, have the potential to result in the administration of more targeted medications at the optimal dosage. The use of technological components, such as digital health platforms and smart delivery devices, is expected to increase adherence and monitoring. There is an increasing interest in the use of apps in the field of pediatrics, especially for dealing with obesity and T2DM in teens [130,131]. Several cost-cutting strategies are currently under investigation. These techniques include creating biosimilar GLP-1RAs and optimizing manufacturing processes. The goal is to improve the availability of these medications. Further research on the safety of GLP-1RAs is currently underway. These studies focus on specific populations and include long-term safety trials and real-world data [132,133]. GLP-1RAs are becoming increasingly important in the treatment of a variety of metabolic and non-metabolic disorders. This suggests that future improvements will lead to more tailored and comprehensive approaches for managing T2DM and obesity.

## Conclusions

The use of GLP-1-RAs has revolutionized the treatment of T2DM and obesity. These medications employ a comprehensive approach, targeting blood sugar regulation, weight loss, and reducing the likelihood of cardiovascular complications. Owing to their distinct method of action, favorable safety profile, and potential for customized therapy, they play a crucial role in combating metabolic diseases and are considered a major addition to the arsenal. The widespread use of GLP-1-RAs in clinical settings can enhance patient outcomes, reduce healthcare expenses, and alleviate the burden on healthcare systems. This is particularly crucial, considering the rising global prevalence of T2DM and obesity. However, to fully exploit the capabilities of these agents, measures must be taken to enhance their availability and affordability, particularly in situations where resources are limited. GLP-1-RAs are expected to be used more efficiently in the coming years due to ongoing research focused on improving formulations, expanding their applications, and ensuring long-term safety. With progress in incretin-based medications, GLP-1-RAs are expected to gain significance in the management of T2DM, obesity, and related ailments.
